# Effects of Dolomitic Limestone on the Properties of Magnesium Oxysulfate Cement

**DOI:** 10.3390/ma17184580

**Published:** 2024-09-18

**Authors:** Juan Camilo Adrada Molano, Adriano Galvão Souza Azevedo, Taís Oliveira Gonçalves Freitas, Gabriela Casemiro Da Silva, Holmer Savastano

**Affiliations:** 1 Department of Biosystems Engineering, University of São Paulo, Pirassununga 13635-900, SP, Brazil; adrianogalvao@usp.br (A.G.S.A.); taisfreitas@usp.br (T.O.G.F.); holmersj@usp.br (H.S.J.); 2 Polytechnic School, University of São Paulo, São Paulo 05508-010, SP, Brazil; gabriela.casemiro@lme.pcc.usp.br

**Keywords:** magnesium oxysulfate cement, active magnesium oxide (a-MgO), standardized hydration method, compressive strength, dolomitic limestone

## Abstract

This study investigated the effects of substituting magnesium oxide (MgO) with dolomitic limestone (DL) on the mechanical and physical properties of magnesium oxysulfate (MOS) cement. Additionally, the hydration formation phases and the influence of the molar ratio on the MOS cement’s performance were examined. The corresponding action mechanisms were identified and explored by compressive strength tests, scanning electron microscopy (SEM), X-ray diffraction (XRD), isothermal calorimetry, and a thermogravimetric analysis (TGA). The results showed that replacing MgO with DL decreased the reaction speed and heat release rate generated in the hydration process of the MOS cement. This substitution also reduced the quantity of non-hydrated MgO particles and delayed the formation of Mg(OH)_2_. The diminished formation of Mg(OH)_2_ contributed to an increase in the apparent porosity of pastes containing DL, thus alleviating internal stresses induced by Mg(OH)_2_ formation and enhancing their mechanical strength after 28 days of curing. Conversely, the increased porosity improved the CO_2_ diffusion within the structure, promoting the formation of magnesium carbonates (MgCO_3_). Through the characterization of the cement matrix (XRD and TGA), it was possible to identify phases, such as the brucite, periclase, and 318 phases. The obtained results revealed the potential of incorporating mineral fillers like limestone as a promising approach to producing MOS cement with a reduced environmental impact and better properties at higher curing ages.

## 1. Introduction

Magnesium oxysulfate (MOS) cement was invented in 1867 and is a type of MgO-based cement similar to Sorel cement, in which magnesium oxide is combined with a magnesium chloride (MgCl_2_) solution to obtain a non-hydraulic binder. Similarly, it is possible to obtain magnesium oxysulfate cement by the reaction between magnesium oxide (MgO) and an aqueous solution of magnesium sulfate (MgSO_4_) [1,2]. This type of non-hydraulic binder has the advantages of being lightweight, having a low level of energy consumption, and providing thermal insulation [3,4]. Additionally, MOS cement exhibits good resistance to acids and alkalis, as well as resistance to saline corrosion, coupled with an excellent bonding performance [2,5,6,7]. As a result, the practical application of MOS cement has been investigated in various areas, including MOS-based concrete, porous insulation materials, fireproof boards, composite panels, decorative panels, prefabricated buildings, and the solidification of heavy metals and solid waste, demonstrating the broad potential of this material in the construction industry [3,5,6,8,9,10].

Another important aspect is that MgO, under appropriate curing conditions, undergoes carbonation reactions due to the absorption of carbon dioxide (CO_2_), thus obtaining a significant improvement in mechanical strength and reducing CO_2_ emissions [11,12]. Similar to the production of ordinary Portland cement (OPC) from limestone, MgO can be produced by calcining magnesite (MgCO_3_) at lower temperatures (1450 °C for OPC and 750 °C for MgO production), which reduces the amount of energy used in the calcination process [10,13]. MgO is not only produced from magnesite calcination but is also synthesized from seawater and brine or waste from the desalination process [14,15]. In addition to its lower energy consumption during production and CO_2_ capture, MgO-based cement has gained attention due to the availability of magnesium ores worldwide. According to the Mineral Commodity Summaries 2022 report, approximately 30,000 tons of compounds were extracted from mines containing magnesium minerals in 2021, with about 2000 tons produced in Brazil during the same period [16]; however, studies on MgO-based building materials are limited, requiring further research to improve their application in the field.

The final properties of MOS cement depend on the hydration products formed after the hardening of the pastes [5]. According to the ternary system of MgO-MgSO_4_-H_2_O, four oxysulfate phases are found at temperatures between 30 °C and 120 °C: (I) 3Mg(OH)2-MgSO_4_-8H_2_O (the 318 phase), (II) 5Mg(OH)_2_-MgSO_4_-3H_2_O (the 513 phase), (III) Mg(OH)_2_-MgSO_4_-5H_2_O (the 115 phase), and (IV) Mg(OH)_2_-2MgSO_4_-3H_2_O (the 123 phase) [17,18,19]. However, among the phases mentioned, the one with the highest chemical stability at temperatures near 23 °C is the 318 phase (3Mg(OH)_2_-MgSO_4_-8H_2_O). This phase presents a better physical/mechanical performance and has better chemical stability, which are essential properties for increasing the durability and mechanical strength of cement, especially MOS cement without chemical additives [18].

The phases with a greater stability depend on the composition of the paste mixture; the molar concentrations of MgO, MgSO_4_, and H_2_O are the main parameters that influence the formation of hydration products [20,21]. Tang et al. [20] evaluated the influence of the MgSO_4_/H_2_O ratio in the pore structure evolution of MOS cement pastes and followed the formation of hydration products by taking non-contact impedance measurements (NCIMs). The results demonstrate that the decrease in the ratio led to a decrease in the kinetics of the formation of hydration products, which were identified by the mass loss in the range between 80 and 250 °C, which is associated with the dehydration of the phases responsible for the strength of the MOS binder. On the other hand, increasing the MgO/MgSO_4_ ratio promoted more significant mass loss and was related to an enhancement in the formation of hydration products (the 517 and 318 phases) [20]. The same authors evaluated the influence of the MgSO_4_/H_2_O ratio on the formation of phases that contribute to the increase in compressive strength of MOS cement samples and the interference in the formation of porosity during the hardening of the samples [21]. The increase in the amount of water during the synthesis of MOS cement promoted a decrease in compressive strength at all curing ages. The MgSO_4_/H_2_O ratio variation caused changes in the pore structure (porosity, incremental pore volume, pore tortuosity, average pore diameter, and maximal pore diameter) of MOS cement pastes.

The use of limestone filler in cementitious materials as a partial substitute has been a standardized practice since the 1980s [22]. It offers several potential environmental benefits because the production of fillers does not require calcination [23]. In contrast, the production of MgO via the calcination of magnesite releases around 3.1–4.5 kg CO_2eq_/kg of MgO, whereas the production of fillers only emits 26–75 g CO_2_/kg of filler [23,24]. In addition, the incorporation of limestone in the matrix contributes to the absorption of the heat released during the exothermic formation of the cement, as well as reducing thermal shocks that may occur [25]. Additionally, it can cause the densification of the microstructure through the filling effect [26]. Furthermore, it has been reported that the addition of limestone has a positive effect on the diffusion of carbon dioxide in cement pastes [27]. Li et al. [28] demonstrated that substituting lime (CaO) for MgO in reactive MgO cement (RMC) resulted in an increased CO_2_ absorption capability under both ambient and accelerated carbonation conditions. While extensive research exists on limestone fillers in Portland cements, there are limited studies on the use of various limestone fillers in magnesium oxysulfate (MOS) cements. Previous research, such as that by Gomes and de Oliveira (2018) [29], has explored the substitution of MgO with carbonates, demonstrating potential improvements in hydration control. In contrast, the current study introduces dolomitic limestone (DL) as an alternative filler in MOS cement. Dolomitic limestone is a low-cost, non-toxic mineral that is infrequently used in Portland cement due to expansion issues associated with the MgO content in DL. Instead, it is commonly employed as an alkalizing agent for pH correction in agricultural applications [30,31]. 

This research aims to assess the feasibility of incorporating DL as a filler in MOS cement formulations. This study involves evaluating MOS cements with varying initial concentrations of MgO, MgSO_4_, and H_2_O and examining the effects of partially replacing MgO with DL. Through comprehensive experimental studies and an analysis, it is expected to develop MOS cement formulations that integrate dolomitic limestone, achieving an optimal functional and mechanical performance while reducing the environmental impact of MOS cement production. 

## 2. Materials and Methods

### 2.1. Raw Materials

**Figure 1:**
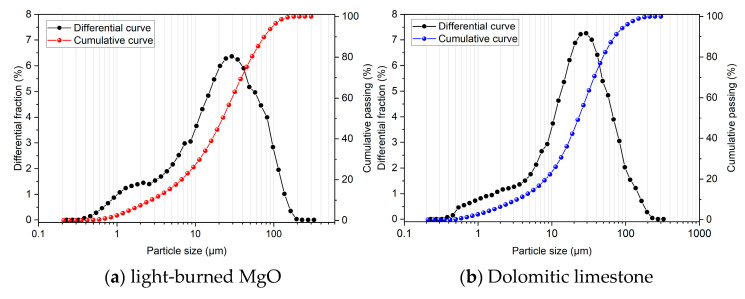
Particle size distributions of the raw materials used.

The light-burned MgO (LBM) used for this work was produced by calcining magnesite at 1050 °C and was provided by RHI Magnesita, Contagem, Minas Gerais State, Brazil. The LBM had a specific surface area of 26.71 m^2^/g (SSA) and a density of 3.45 g/cm^3^. The analytically pure magnesium sulfate heptahydrate (commercial-grade Epsom salt, MgSO_4_·7H_2_O, Labsynth, Diadema, Brazil) used in this study was purchased with a purity of 99,83%. The dolomite limestone (DL), provided by Infibra, located in Leme, São Paulo, Brazil, had a density of 2.79 g/cm^3^. The chemical compositions and size distributions of the LBM and DL given in Table 1 and Figure 1 were obtained using X-ray fluorescence (XRF, Malvern Panalytical, Malvern, UK, Zetium model) and a laser scattering particle analyzer (Horiba, Kyoto, Japan, LA-950), respectively. LBM and DL’s median particle sizes (D50) were 22.20 µm and 23.10 µm, respectively.

We used the standard hydration test, a Chinese methodology standardized by the WB/T1019 (2002) [32], to determine the content of active MgO (α-MgO). According to Dong et al. [33], only active magnesium oxide can react with water to form Mg(OH)_2_. Based on this perspective, we can determine the content of α-MgO in magnesium oxide by Equation (1), and the active magnesia content of LBM powder in this study was approximately 63%.

(1) $$\alpha -MgO=\frac{40\left(m_{2}-m_{1}\right)}{18m_{1}}$$

where m_1_ is the mass (g) of LBM before reacting with water, and m_2_ is its mass (g) after reacting with water for three hours at 105 °C, respectively.

The experiment was conducted using 125 mL of deionized water. The system was heated to 80 °C, and then 2.5 g of LBM was added. The solution was homogenized using a magnetic stirrer, and a cover was utilized to prevent evaporation, ensuring minimal water loss during the process. The hydration times were 10, 20, 30, 40, 60, 90, 120, 150, and 180 min to study the hydration kinetics of the MgO. Posteriorly, the hydrated material was filtered and washed repeatedly with anhydrous ethanol. The products were dried at 105 °C for 24 h to remove all water that had not been chemically combined. Based on Equation (1), the weight of the prepared sample was used to calculate the hydration rate of MgO.

### 2.2. Specimen Preparation

The different MOS cement mixtures were prepared mixing MgO, DL, MgSO_4_·7H_2_O, and H_2_O. The MgSO_4_·7H_2_O was dissolved in deionized water at approximately 60 °C, resulting in a solution with a 25% (*w*/*v*) concentration of MgSO_4_. The temperature of 60 °C was selected to enhance the solubility of MgSO_4_, ensuring complete dissolution and uniformity of the solution. Lower temperatures were insufficient for complete dissolution. The necessary amount of MgO powder was homogenized with dolomitic limestone for 1 min. Then, it was added to the solution and blended for four minutes to prepare MOS cement pastes. Various MOS cement pastes with different MgO:MgSO_4_:H_2_O molar ratios were prepared. The mixing proportions are given in Table 2.

The water–cement ratio (W/C) of the MOS cement pastes was calculated according to Equation (2). In this equation, M_H2O_, M_MgO_, M_MgSO4_, and M_DL_ are the weights of water in magnesium sulfate solution (75% by weight), light-burned MgO, magnesium sulfate anhydrous in magnesium sulfate solution (25% by weight), and DL, respectively.

(2) $$\frac{W}{C}=\frac{M_{H2O}}{M_{MgO}+M_{MgSO_{4}}+M_{DL}}$$

Fresh pastes were cast into 50 mm × 25 mm (height × diameter) cylindrical molds. During the first 24 h, the specimens were placed in sealed plastic bags and kept at room temperature. This sealed environment helps prevent moisture loss and ensures that the paste remains properly hydrated.

After this initial period, the specimens were transferred to a climate chamber with a relative humidity of (60 ± 5)% and a temperature of (23 ± 2) °C until they reached the ages of analysis, which are 7 and 28 days. A total of 6 specimens were used for each condition.

### 2.3. Analytical Methods

In this work, according to ASTM C807 [34], the setting time of the MOS cement pastes was performed by Vicat needle. For the setting time tests, each paste was tested using a single measurement with a substantial volume of mixture to ensure consistency and reduce potential variations from the mixing and molding process. However, for a more robust statistical analysis, it would be ideal to conduct multiple tests for each mixture. Apparent porosity was determined after the specimens were cured in a climate chamber with a relative humidity of (60 ± 5)% and a temperature of (23 ± 2) °C for 7 days. After this period of controlled curing, the apparent porosity was measured in accordance with ASTM C150 [35]; the calculation is performed according to Equation (3).

(3) $$I_{v}=\frac{{m_{sat}-m}_{s}}{m_{sat}-m_{i}}$$

where *m_sat_* is the mass of the sample saturated in water after immersing and boiling it (g), *m_s_* is the sample dried in the oven at (105 ± 5) °C for 72 h, and *m_i_* is the mass of the sample immersed in water (g).

The compressive strength test of MOS cement paste at 7 and 28 days was carried out following ASTM C109 [36]. The testing was performed using EMIC DL 30000 (Instron, Paraná, Brazil), and the deformation rate was set at 0.3 mm/min.

The hydration reactions of MOS cement pastes were monitored using isothermal calorimetry, X-ray diffraction, thermogravimetry, and scanning electron microscopy. Heat release during hydration of MOS cement pastes was quantified using isothermal calorimetry with TAM Air (TA Instruments, New Castle, DE, USA) with a precision of ±20 μW for 72 h. Data collection started 4 min after the LBM and DL were added to the MgSO_4_ solution. The crystalline composition was identified by X-ray diffraction (XRD) on powdered samples using a Rigaku Rotaflex^®^ Mini Flex 600 diffractometer (Tokyo, Japan). The equipment was operated with a scanning range of 5 to 65° (2θ), scanning at a rate of 10°/min with steps of 0.02°. The analysis was conducted with a voltage of 50 kV and an electric current of 100 mA. Thermal decomposition of the hydrated products was measured using a Libra 209 F1 thermobalance (Netzsch, Selb, Germany) under a nitrogen atmosphere, from 30 °C to 1000 °C at a heating rate of 10 °C per minute. Scanning electron microscope (SEM) images were obtained using a Hitachi TM3000 microscope (Tokyo, Japan), and energy-dispersive X-ray spectroscopy (EDS) measurements were conducted with a SwiftED3000 (Oxford Instruments, Abingdon, UK). The SEM analysis was carried out at an acceleration voltage of 15 kV.

## 3. Results and Discussion

### 3.1. Effects of Material Ratio and DL on the Setting Time of MOS Cement

**Figure 2:**
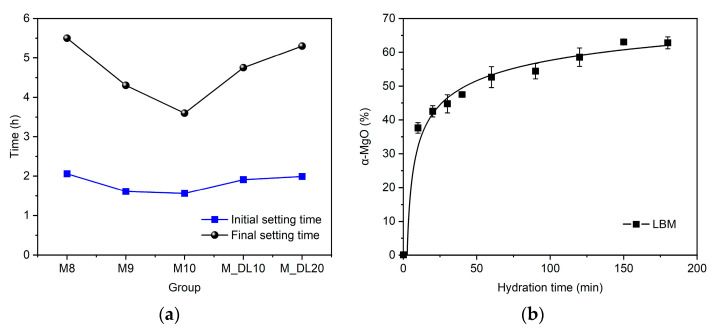
Setting time of MOS cement pastes (**a**) and hydration kinetics of light-burned MgO (LBM) (**b**).

Figure 2 shows the setting time of MOS cement pastes with different molar ratios of MgO/MgSO_4_ and DL contents. It can be seen from the picture that increasing the molar ratio significantly decreases the final setting times of the pastes. For ratios of nine and ten (M9 and M10 samples), the final set times were decreased by 22% and 34%, respectively, when compared to the M8 mixture. However, for the initial setting times, the increase in molar ratio did not have a significant effect on the hydration. This is due to the hydration process of magnesium oxide; as observed in Figure 2b, the hydration kinetics of MgO exhibit a rapid initial increase followed by a deceleration. After approximately 120 min of hydration, a phase of stability in the hydration reactions is reached, corresponding to the initial setting times of the compositions [37], when the surface of the MgO particles is converted into magnesium hydroxide phases [38]. The isothermal calorimetry for the MOS cement, discussed in Section 3.5, shows the Mg(OH)_2_ formation in the first stages of the hydration process (pre-induction–induction), which occurs in the first 2 h, showing the relationship between a-MgO and the initial hydration time.

Furthermore, increasing the amount of dolomitic limestone as a replacement for MgO substantially increased the final setting time, although it did not significantly change the initial setting time of the mixtures with DL. Overall, a lower MgO content in the composition of the pastes delayed and prolonged the hydration process and the final setting time in the MOS cement, while the initial setting time of the pastes depends mainly on the active MgO content and the hydration kinetics for pastes with the same MgSO_4_/H_2_O molar ratio [18]. The delaying effect of replacing MgO with DL may influence the development of early strength values [39], as will be examined and discussed in detail in Section 3.2.

### 3.2. Compressive Strength of MOS Cement Pastes

**Figure 3:**
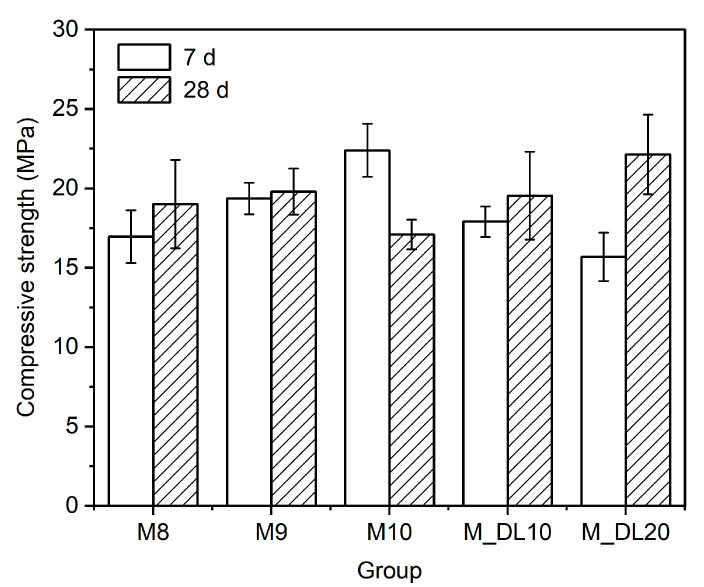
Compressive strength of the MOS cement pastes cured for 7 and 28 days.

Figure 3 shows the compressive strength of the MOS cement samples at 7 and 28 days of curing, prepared with different molar ratios of MgO/MgSO_4_ (M8, M9, and M10) and with the replacement of MgO with DL at 10% and 20% by weight in the M10 sample (M_DL10 and M_DL20). After seven days of controlled curing, the compressive strength of the MOS cement pastes is enhanced with increasing molar ratio, by 14% and 32% for the M9 and M10 samples, respectively, compared to M8. However, increasing the replacement of MgO with DL decreased the compressive strength of M10 by 20% and 28%, when the MgO was replaced with 10% and 20% DL, respectively.

The mechanical performance showed a different behavior at 28 days of curing. For the samples without DL, a higher molar ratio resulted in a less gradual increase in compressive strength, until a loss of strength for the M10 sample was attained, with a 23% decrease at 7 days of curing. This is attributed to the formation of the hydration phases of the MOS cement, which is discussed in the mechanism analysis in Section 3.3. For the pastes with DL replacement, contrary to the mechanical results obtained at seven days of curing, increasing the replacement of MgO by DL improved the compressive strength of the MOS cement at 28 days.

At longer curing times (28 days) for pastes with the same MgSO_4_/H_2_O molar ratio, the lower amount of MgO in the compositions resulted in increased compressive strength, particularly when a lower MgO/MgSO_4_ molar ratio was used. This effect is most pronounced when comparing pastes with MgO replaced with dolomitic limestone (DL), specifically M_DL10 and M_DL20, with the M10 sample. The incorporation of DL initially delayed the mechanical strength gain, but it ultimately contributed to a higher compressive strength at later curing ages. In summary, the inclusion of DL in pastes is advantageous for improving their compressive strength over extended curing periods.

### 3.3. X-ray Diffraction Analysis (XRD)

**Figure 4:**
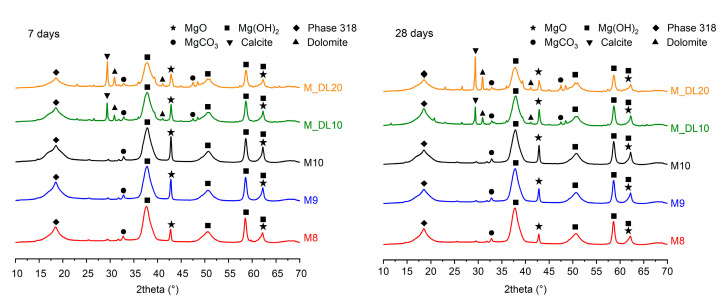
Diffractograms of the MOS cement pastes cured for 7 (**left**) and 28 (**right**) days.

The effect of the molar ratio and incorporation of DL on the formation of the hydration phases of the MOS cement, hardened at the ages of 7 and 28 days, is presented in Figure 4. The results show that the main hydration products of the mixtures are composed of the 318 phase (3Mg(OH)_2_∙MgSO_4_∙8H_2_O), brucite (Mg(OH)_2_) and periclase (MgO), and smaller amounts of amorphous phases of dolomite (CaMg(CO_3_)_2_), magnesite (MgCO_3_), and calcite (CaCO_3_). The presence of MgCO_3_ in the pastes does not change significantly at different curing times and is attributed to the carbonation of the surface of the pastes [20]. CO_2_ sequestration was most evident for the samples with DL, promoting the formation of new MgCO_3_ peaks at around 47.4 degrees. One of the main crystallized hydration products in MOS cement pastes, which contribute to the mechanical strength gain, is the 318 phase; however, recent research has identified that the 318 phase is metastable at room temperature [40], and diffractograms show that the peak intensity of the phase at an angle of 18.5 at seven days increases with the increment of the molar ratio of MgO/MgSO_4_, contributing to the strength gain. However, at 28 days, the peak intensity of the 318 phase decreased in the M10 paste, resulting in a decrease in compressive strength, as presented in the mechanical performance results. This decrease suggests that the instability of the 318 phase under environmental conditions may have led to its transformation or partial decomposition, reducing its presence and, consequently, compressive strength. The hydration phase’s instability contributes to a decreased compressive strength at longer curing times in MOS cement prepared without chemical additives. In addition, the peak intensities of unhydrated MgO at 42.8 and 62.2 degrees decreased between 7 d and 28 d. This may be caused by the formation of Mg(OH)_2_ [41]. Similarly, the presence of unhydrated MgO can promote the late formation of brucite in conditions of high humidity [42]. On the other hand, the substitution of MgO by DL decreased the amount of unhydrated MgO at 7 d and Mg(OH)_2_ formation at 28 d and promoted the formation of new amorphous phases of dolomite and calcite at around 30.9 and 29.3 degrees. The smaller formation of brucite at early curing ages resulted in the lower mechanical strengths at 7 d of the samples with DL. However, the filling effect of the DL particles [26], the smaller formation of late brucite, and the formation of MgCO_3_ and the 318 phase possibly can be attributed to the increased compressive strength at higher curing ages in the M_DL10 and M_DL20 samples compared to M10.

### 3.4. TG/DTG Analysis

**Figure 5:**
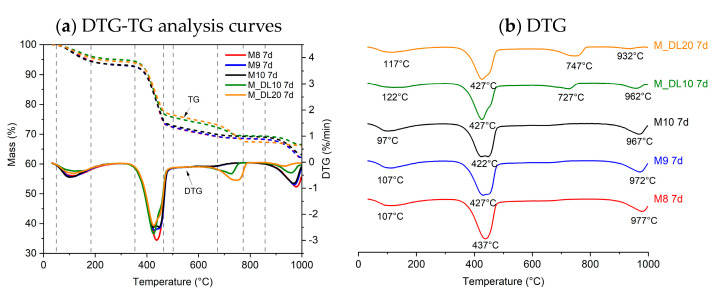
Thermogravimetric curves of MOS cement pastes after 7 days of air curing.

**Figure 6:**
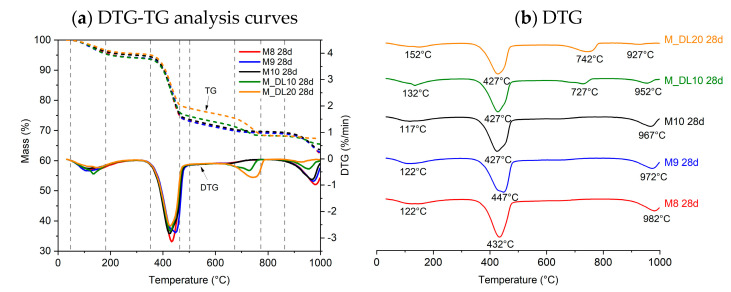
Thermogravimetric curves of MOS cement pastes after 28 days of air curing.

The thermogravimetric analysis (TGA) was conducted on the MOS cement pastes after 7 and 28 days of hydration, as shown in Figure 5 and Figure 6, respectively. From the derivative thermogravimetric (DTG) curve, it is possible to more clearly identify the temperature ranges corresponding to the decomposition of the phases present in MOS cement. The first mass loss, in the range of 50–180 °C, corresponds to the dehydration of the 318 phase due to the loss of adsorbed water. Subsequently, between 350 and 460 °C, the dehydroxylation of uncarbonated brucite or brucite within hydrated magnesium carbonates (HMCs) occurs. The decomposition of carbonates, such as MgCO_3_ and CaCO_3_, and HMCs results in endothermic peaks in the range of 650–900 °C and is observed in the mixtures to which dolomitic limestone was added. Above 900 °C, the desulfurization process of MgSO_4_ in the 318 phase occurs. It can be seen that the samples containing the dolomitic filler showed less desulfurization, which may be associated with the lower amount of MgSO_4_ present in the hydration phases. The formation of MOS occurs by MgO reacting with H_2_O and forming brucite on the surface of the MgO particles: MgO + H_2_O → Mg(OH)_2_. This brucite, when its concentration on the surface of the particles increases, migrates to the MgSO_4_ solution: 3Mg(OH)_2_ + Mg^2+^ + SO_4_^2−^+ 8H_2_O → 3Mg(OH)_2_-MgSO_4_-8H_2_O (318 phase). When DL was added to replace MgO, the formation of brucite and Mg^2+^ and SO_4_^2−^ decreased, which was reflected in the lower mechanical strength at 7 days of the M_DL10 and M_DL20 samples [27,43].

The thermogravimetric analysis did not show significant changes with increasing MgO/MgSO_4_ molar ratio, although the mass loss related to MgSO_4_ decomposition appears to decrease with increasing MgO content. With the progression of hydration from 7 to 28 days, it was noted that MgO hydrates slowly during this period to form brucite, which is consistent with the reduction in the intensity of the residual MgO peak in the XRD analysis. The increase in mass loss due to brucite dehydroxylation at 28 days is also associated with a slight increase in MgCO_3_ mass loss and the formation of a ripple in the thermogravimetric curve profile in the range of 350–460 °C. This waviness, also reported in the literature, is correlated with the dehydroxylation of the brucite present in the structure of HMCs [27,44,45].

**Figure 7:**
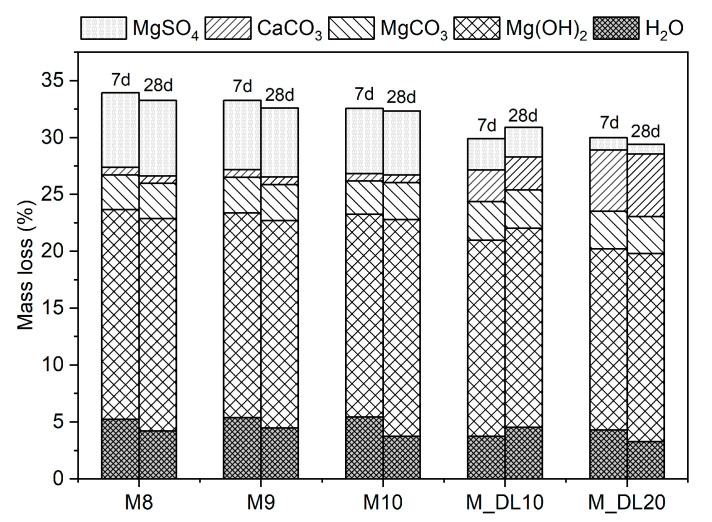
Mass loss of each component of MOS cement pastes at 7 and 28 days of air curing.

The replacement of MgO by DL led to a reduction in the formation of brucite and of the 318 phase and promoted the formation of mineral phases such as dolomite and calcite, as shown in the XRD analysis. This is possible to verify in the DTG by noting the appearance of a peak related to the decarbonization of MgCO_3_ and CaCO_3_. Increasing the replacement of MgO with DL from 10% to 20% reduced MgSO_4_-related mass loss by 61.6% and 67% at 7 and 28 days of hydration, respectively, as observed in Figure 7. This reduction was reflected in the loss of strength at seven days as previously reported; however, this was compensated for at 28 days by the filling effect of the dolomitic limestone, which was enhanced through the hydration process. 

### 3.5. Hydration Heat Evolution

**Figure 8:**
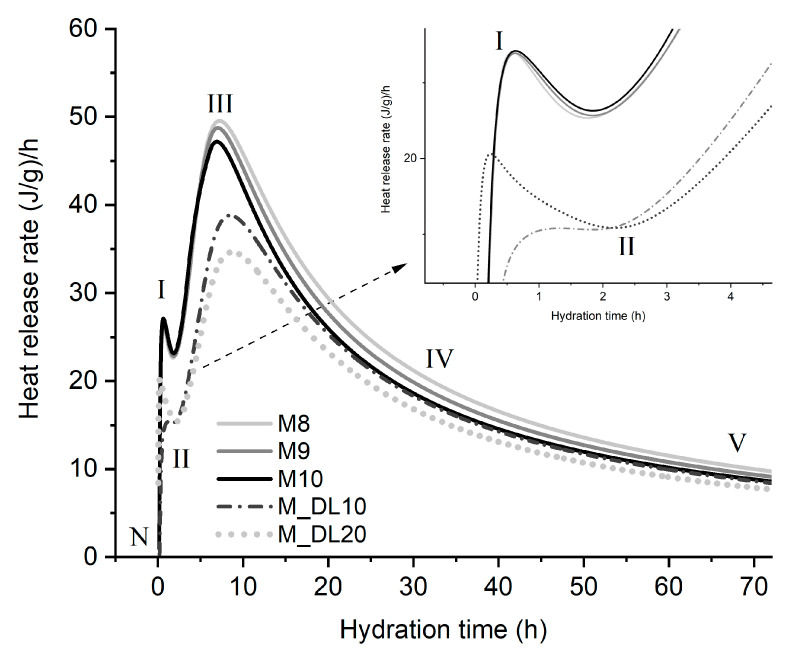
Isothermal calorimetry curves for MOS cement pastes.

**Figure 9:**
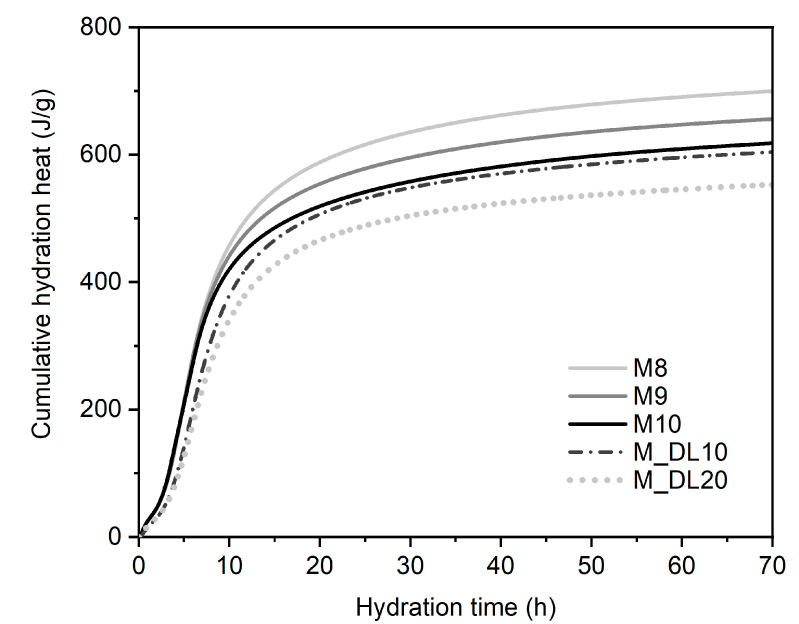
Cumulative hydration heat of MOS cement pastes.

The 72 h hydration heat evolution and accumulative heat of the MOS cement with the different molar ratios of MgO/MgSO_4_ and levels of replacement of MgO with DL were measured by isothermal calorimetry, and the results are shown in Figure 8 and Figure 9, respectively. According to the heat release rate of hydration of the MOS cement, the hydration process can be divided into five stages: pre-induction (N–I), induction (I–II), acceleration (II–III), deceleration (III–IV), and a stable period (IV–V) [18,38,46]. The pre-induction period occurs at the beginning of the mixing of the active MgO with the aqueous magnesium sulfate solution; it is observed that during this period, M8, M9, and M10 do not have a significant difference in the rate of heat released; however, the inclusion of DL as a substitute for MgO decreased the amount of heat released, which corresponds to a lower amount of OH^−^ generated [18]. The insoluble Mg(OH)_2_ formed on the surface of the MgO particles prevented the different hydration reactions to some extent, which led to a decrease in the heat of hydration shown in the induction period. Similar to the first period, the rate of heat released was up to 37% lower when the DL was incorporated compared to that of M10. The hydration time until the end of the period was approximately two hours for all of the samples; after this time, the rate of hydration heat release accelerated rapidly, starting with the acceleration period, related to the initial setting time of the pastes, as seen in Figure 2a, and the formation of hydration products composed mainly of the 318 phase and Mg(OH)_2_ [38]. The isothermal peaks shown in stage III show a gradual reduction in heat release rates when the molar ratio increases and are more evident when the substitution rate of MgO with DL is higher, approximately by 34% and 40% less for the pastes of M_DL10 and M_DL20, respectively, compared to M10. After reaching the highest heat release value, the hydration process enters the deceleration period due to the decrease in the diffusion rate and ion concentration, the hardening of the paste, and the lower surface area of the active MgO, until it reaches a stable period. In summary, as can be seen in Figure 9 and Table 3, increasing the molar ratio and replacing MgO with DL decreased the cumulative heat of hydration of the MOS cement.

**Figure 10:**
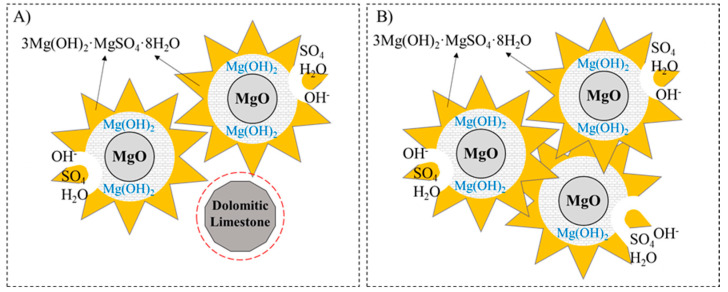
The hydration mechanism of MOS at acceleration period with (**A**) and without dolomitic limestone (**B**). Adapted from [38].

In addition to the decreasing heat release rates at all stages, as shown in Table 4, the dolomitic limestone slightly delayed the hydration of MgO and the formation of the phases (the 318 phase) in the MOS cement during the acceleration period, which may have influenced the lower compressive strength values obtained at the earliest curing ages (7 d). The behavior observed in this work can be partially explained by the fact that a more significant substitution rate of MgO with DL decreased the number of hydration cores of active MgO, replacing them with non-hydrated dolomite and calcite products, as observed in the XRD analysis. Consequently, the reaction speed and total heat rate of the MOS cement phases decreased during hydration, as shown in Figure 10.

### 3.6. Microstructural Analysis

**Figure 11:**
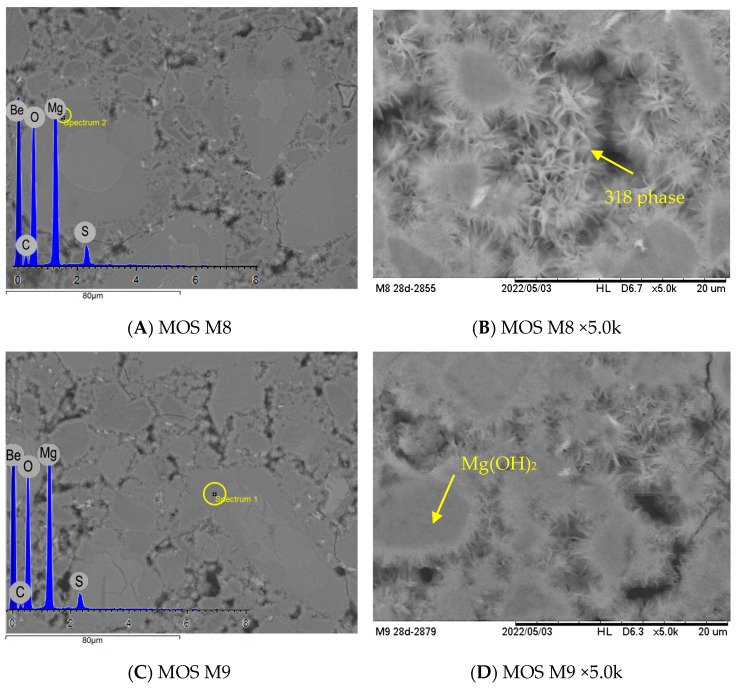

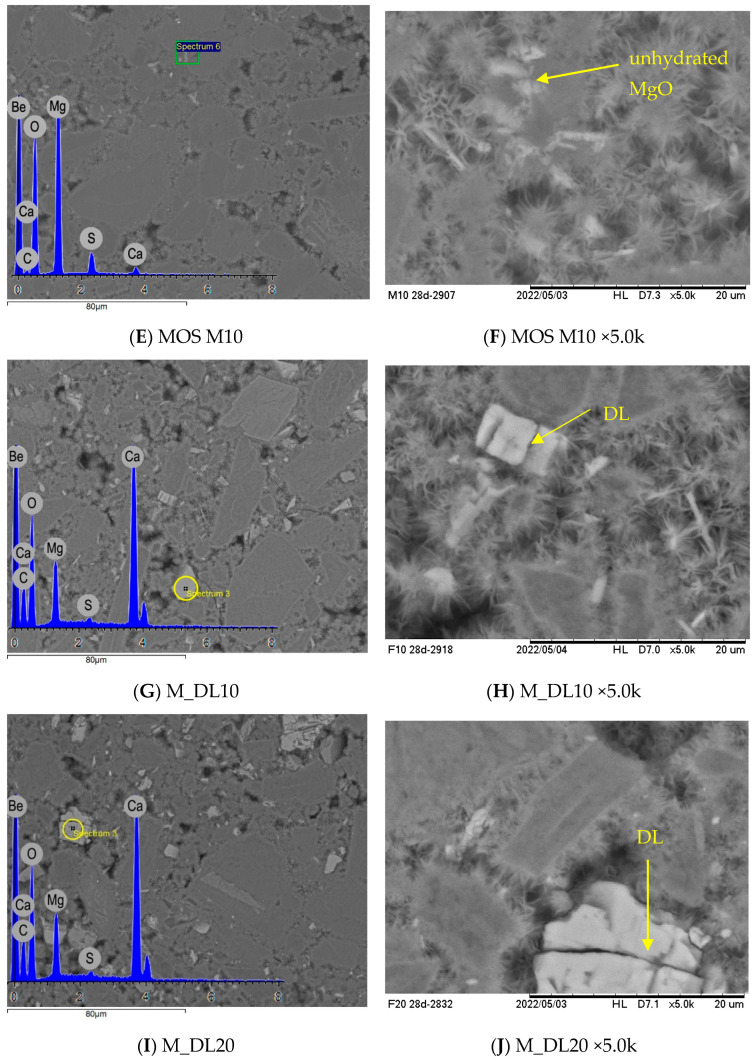
Fractured surface morphology of MOS samples after 28 days of curing.

**Figure 12:**
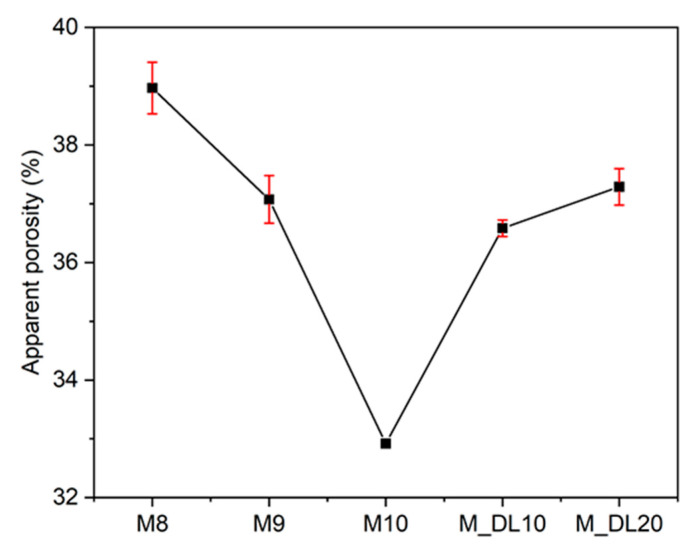
Apparent porosity of MOS cement pastes after curing for 7 days.

Figure 11 shows the SEM images of the MOS cement samples at 28 d of curing. The XRD and EDS results indicate that the plate-like crystals are the Mg(OH)_2_ phase, while the 318 phase starts to form and grow at the solid–liquid interface of the Mg(OH)_2_ crystals, as described in Section 3.5. According to Huihui Du et al. (2022) [38], the needle-cluster phase in MOS cement is considered to be the 318 phase, which is responsible for the increase in strength at longer curing times, while the Mg(OH)_2_ contributes to the initial strength gain [38,40]. It is also observed in Figure 11F that a large amount of unhydrated MgO particles could explain the lower heat of hydration generated in M10 compared to M8 and M9; some hydrated products gradually cover these particles that are present in all samples. The higher compressive strength results in the M_DL10 and M_DL20 matrix (Figure 3) may be due to the needle-cluster crystals of the 318 phase that seem to have a higher growth at the solid–liquid interface at 28 d, compared to the M10 matrix, which shows the greater formation of the Mg(OH)_2_ plates and lower levels of porosity, as observed in Figure 11E and Figure 12. On the other hand, the DL particles showed no formation of crystallization phases on their surface, and it can be observed in the microstructures shown in Figure 11E,G,I that the porosity increases with the increase in the replacement rate of MgO with DL, which was also confirmed by the apparent porosity tests after seven days of curing (Figure 12). These observations may explain the formation of the new MgCO_3_ peaks in the M_DL10 and M_DL20 pastes, due to a better diffusion of CO_2_ in the MOS cement structure promoted by the higher level of porosity [47]. These observations are consistent with the mechanical results, as well as the XRD and isothermal calorimetry analyses.

However, the porosity results at 28 days were not presented due to problems encountered during the experimental procedure. Specifically, when the samples were dried at 105 °C for 24 h, cracks occurred on the surface. In addition, after immersing the samples in water for 72 h, more cracks were observed, due to the high formation of Mg(OH)_2_, which caused volumetric expansion and increased instances of cracking, which were particularly evident at 28 days.

## 4. Conclusions

In this study, MOS cement with different molar ratios of MgO/MgSO_4_ was used to evaluate the effects of dolomitic limestone on its mechanical and physical properties and the formation of hydration phases. The corresponding action mechanisms were identified and explored in terms of their hydration mechanism, XRD analyses, SEM results, and others. The obtained results revealed that the increase in porosity in the microstructure of MOS cement pastes due to the replacement of MgO with dolomitic limestone could alleviate the internal stresses produced by the expansion of MgO hydration during air curing and improve the pastes’ long-term mechanical strength values. Moreover, it enhances CO_2_ diffusion in the cement MOS structure, promoting the formation of magnesium carbonates (MgCO_3_). In addition, based on the above investigations, we can draw the following specific conclusions:

- The substitution of MgO with dolomitic limestone decreases the heat release rate generated, prolongs the hydration process of the MOS cement, and reduces the amount of unhydrated MgO and late Mg(OH)_2_ formation;
- The decreasing content of the hydration product, the 318 phase, and greater brucite formation at longer curing times decrease the mechanical strength of MOS cement prepared without chemical additives;
- A lower MgO content in the paste composition delays and prolongs the hydration process and final setting time in MOS cement, while the initial setting time of the pastes, for an equal molar ratio of MgSO_4_/H_2_O, depends mainly on the content of active MgO and the hydration kinetics of LBM;
- Microstructural analyses demonstrate the formation of 318 phase crystals, as well as the presence of dolomitic limestone embedded within the cementitious matrix. This can be associated with the filler effect, which results in the improved physical and mechanical performance of the materials after extended curing periods.

Based on this work, it is suggested that future work be carried out in this line of research in order to better study the durability and effect of accelerated carbonation curing on MOS cement matrices with higher levels of porosity using calcareous fillers.

## Figures and Tables

**Table 1:** Chemical compositions of LBM and DL.

Samples	Mass Fraction (wt. %)									
	MgO	Al_2_O_3_	SiO_2_	SO_3_	Cl	K_2_O	CaO	MnO	Fe_2_O_3_	LOI *
LBM	94.1	0.09	0.28	0.26	0.06	-	1.47	0.18	0.91	2.62
DL	7.60	1.03	3.99	0.13	-	0.22	43.9	0.09	0.42	42.4

* Loss on ignition at 1020 °C.

**Table 2:** Mixing proportions of MOS cement pastes.

Mixtures	Molar Ratio MgO:MgSO_4_:H_2_O	Dolomitic Limestone (% by Weight of MgO)	W/C Ratio
M8	8:1:20	-	0.70
M9	9:1:20	-	0.64
M10	10:1:20	-	0.59
M_DL10	10:1:20	10	0.59
M_DL20	10:1:20	20	0.59

**Table 3:** Summary of the isothermal calorimetry test results.

Mixture	The Maximum Heat Release Rate	Hydration Time	72 h Cumulative Heat
	(J/g)/h	(h)	(J/g of MgO Normalized)
M8	49.55	7.2	701.14
M9	48.74	7.03	657.41
M10	47.18	6.92	619.6
M_DL10	38.81	8.47	605.66
M_DL20	34.64	8.76	554.06

**Table 4:** Effect of dolomitic limestone (DL) on the heat release rate of MOS cement.

Period	Average Heat Release Rate Decrease (%) *	
	M_DL10	M_DL20
(N–I)	51	20
(I–II)	37	31
(II–III)	34	40
(III–IV)	13	22
(IV–V)	2	10

* Compared to the M10 sample.

## Data Availability

The original contributions presented in the study are included in the article, further inquiries can be directed to the corresponding author.
